# Effect of Tween 20 and 80 Addition During the Chopping Process on Gel Quality of Silver Carp Surimi

**DOI:** 10.3390/foods15142544

**Published:** 2026-07-18

**Authors:** Yu Zhang, Yulong Bao, Yuemei Zhang, Yi-Ming Zhao

**Affiliations:** 1 School of Food Science and Engineering, Jiangsu University, Zhenjiang 212013, China; zhangyu_zy2024@163.com (Y.Z.); zhaoyiming1129@126.com (Y.-M.Z.); 2 Key Laboratory of Geriatric Nutrition and Health (Beijing Technology and Business University), Ministry of Education, Beijing 100048, China; zhangyuemei@btbu.edu.cn

**Keywords:** surimi, protein denaturation, forward extrusion, myofibrillar protein, surfactant

## Abstract

The impact of nonionic surfactant (Tween 20 and Tween 80) addition during the chopping process on the surimi gel quality was investigated. Results showed that the addition of Tween 20 or Tween 80 significantly decreased myofibrillar protein solubility in a dose-dependent manner. The surface hydrophobicity of myofibrillar proteins increased, likely due to the binding of surfactants. However, the added surfactants exerted a protective effect on myofibrillar protein denaturation, indicated by the greater intensity of intrinsic fluorescence and a higher proportion of ordered secondary structure. Forward-extrusion tests demonstrated that the surfactants reduced the cohesion and adhesion of the surimi paste, leading to improved uniformity and easier extrusion. For the heat-set surimi gel, addition of Tween 20 and Tween 80 led to a less compact, more porous microstructure, which ultimately led to reduced gel strength, hardness, and water-holding capacity. These findings showed that Tween 20 and Tween 80 can protect proteins against denaturation during chopping and improve the flowability of surimi paste, and the weakened mechanical strength of heat-set gel offered potential applications in developing specialized textured foods for the elderly.

## 1. Introduction

Protein denaturation is essential for the texture formation of surimi products. During the heat-set gelation of surimi products, myofibrillar proteins, especially myosin, unfold to interact and form a continuous network [1]. However, excessive denaturation prior to the gelation process will lead to reduced gelling ability [2,3]. Therefore, one major effort in the surimi industry is to minimize the protein denaturation during manufacturing and storage of raw surimi. Freezing is the most common method for the storage of surimi; various additives, such as polysaccharides [4,5], disaccharides [6], protein hydrolysates [7,8] and polyphenols [9,10] have been incorporated to reduce the formation of ice crystals and hence prevent freezing-induced protein denaturation [11]. Chopping is another critical step that influences protein functionality in surimi. It is well known that the chopping temperature should be maintained below 10 °C as the local temperature may rise, which leads to protein denaturation in chopping. When using non-vacuum equipment, the blending of air into the meat batter introduced an air–water interface. It is known that proteins can be denatured by adsorption on interfaces, especially hydrophobic ones [12]. The hydrophobic interface is frequently encountered in surimi products with added oil [13,14]. Protein denaturation at interfaces has been extensively studied, and it has been reviewed with regard to different interfaces, including oil–water [15], ice–water [16], air–water [17], etc.

Proteins also experience shear stress during chopping. It was demonstrated that high shear force or high shear rate alone only led to slight conformational changes [18]. The same authors suggested that the presence of an air–liquid interface may play a role, and therefore they investigated the combined effects of shear and interface on the denaturation of recombinant human growth hormone (rhGH) and recombinant human deoxyribonuclease (rhDNase). The results showed that aggregation of rhGH only occurred in the presence of an air–liquid interface, and the extent of aggregation increased at higher shear rates. When surfactants were used, aggregation of rhGH significantly reduced, likely due to the competitive occupation of the interface by the surfactant. Similarly, Chou et al. [19] demonstrated that Tween 20 and Tween 80 can protect albutropin against agitation-induced aggregation. According to Chou et al. [19], the potential mechanisms for protein stabilization by surfactants include: (1) nonionic surfactants can compete with proteins for adsorption sites on interfaces, (2) nonionic surfactants can bind to hydrophobic regions of the protein surface and thus decrease intermolecular interaction, (3) nonionic surfactants can bind to proteins and increase the free energy of protein unfolding, and (4) nonionic surfactants may act as a chemical chaperone, favoring refolding over aggregation. Other than the protection effects, surfactants are known to unfold proteins depending on the surfactant’s properties (e.g., charge and hydrophobicity), protein characteristics (e.g., charge, shape, molecular weight), and solution conditions (e.g., pH, ionic strength, and temperature) [20].

Both Tween 20 and Tween 80 are widely used nonionic surfactants in foods. According to the European Food Safety Authority, the acceptable daily intake for Tween 20/80 is established at 25 mg/kg body weight per day [21]. The fatty-acid difference (lauric acid chain on Tween 20, oleic acid chain on Tween 20) changes the solubility, foaming behavior, and taste profile. Jakubczyk et al. [22] demonstrated that adding Tween 20 in the 0.10 to 0.70% range to agar gel can be a factor in obtaining a tailored structure and texture. Goff et al. [23] used 0.08% (*w*/*w*) tween 80 in an ice cream mix. At this level, the emulsifier effectively reduced the fat-serum interfacial tension and significantly decreased casein micelle adsorption onto fat globules, thereby promoting partial fat destabilization during freezing without causing excessive churning. Tween 80 has been used in minced meat to stabilize nano-emulsions [24], which deliver antioxidants and antimicrobial compounds efficiently. In the presence of multiple emulsifiers, Yu et al. [24] found that the addition of Tween 80 to emulsified surimi led to a competitive displacement of sodium caseinate and ovalbumin, due to the low interfacial tension of Tween 80. However, the interaction between myofibrillar proteins and the surfactants (Tween 20 and Tween 80) in surimi is not known. The aim of this study was to investigate how the addition of Tween 20 or Tween 80 influences the physicochemical properties of myofibrillar proteins in the surimi and subsequently affects the surimi gel quality.

## 2. Materials and Methods

### 2.1. Materials

Frozen surimi made from silver carp (*Hypophthalmichthys molitrix*) was obtained from Honghu Jingli Aquatic Products Food Co., Ltd. (Honghu, China). SYPRO orange dye (5000×) was purchased from supelco, a brand of Sigma-Aldrich Trading Co., Ltd. (Shanghai, China). All chemicals were of analytical grade and purchased from Sinopharm Chemical Reagent Co., Ltd. (Shanghai, China).

### 2.2. Characterization of Protein in the Raw Surimi

#### 2.2.1. Solubility of Myofibrillar Protein

Raw surimi meat (5 g) was mixed with 20 mL of a solution containing 0.10 M NaCl and 20 mM PBS (3.30 mM Na_2_HPO_4_, 16.70 mM NaH_2_PO_4_, pH 6.5) and homogenized at 8400 r/min for 90 s. The resulting homogenate was centrifuged at 8000× *g* for 15 min at 4 °C. The supernatant was discarded, and the precipitate was washed twice with the aforementioned buffer. The precipitate was then resuspended in 20 mL of a solution containing 0.60 M NaCl and 20 mM PBS (pH 6.5) and centrifuged at 8000× *g* for 15 min at 4 °C. Myofibrillar protein solubility was calculated as the percentage of myofibrillar protein in the final supernatant according to Shen et al. [25].

#### 2.2.2. Intrinsic Fluorescence Spectroscopy

Intrinsic fluorescence spectroscopy was determined using the method of Monto et al. [13] with minor modifications. Fluorescence spectra of myofibrillar protein were measured using a fluorescence spectrophotometer (LengGuang Tech., Shanghai, China). The protein was diluted to 0.1 mg/mL with 20 mM PBS (pH 6.5) containing 0.60 M NaCl. Fluorescence spectra were recorded with the following parameters: an excitation wavelength of 283 nm, an emission range of 300–450 nm, and a slit width of 2.5 nm.

#### 2.2.3. Raman Spectroscopy

Myofibrillar proteins were freeze-dried and then analyzed using high-resolution Raman spectroscopy (DXR Raman Spectrometer, Thermo Fisher Scientific, Madison, WI, USA) according to Yan et al. [26]. The wavelength range was measured from 400 to 3200 cm^−1^. Baseline correction and normalization were performed using Omnic V8.1 software (Thermo Fisher Scientific, Madison, WI, USA). The secondary structure fractions, including α-helix, β-sheet, β-turn, and random coil, were calculated using PeakFit 4.12 software (Systat Software, San Jose, CA, USA) according to Zhang et al. [27].

#### 2.2.4. Surface Hydrophobicity

Surface hydrophobicity was measured using the bromophenol blue (BPB) binding method [28] with modifications. Briefly, 1 mL of myofibrillar protein suspension (5 mg/mL in 20 mM PBS, pH 6.5) was mixed with 0.2 mL of BPB (1 mg/mL). For the control, 1 mL of PBS solution (20 mM) was mixed with 0.2 mL of BPB. After vortexing for 30 min and centrifugation at 8000× *g* for 10 min, the supernatant was diluted 10-fold, and the absorbance was measured at 595 nm. Surface hydrophobicity was expressed as the mount of bound bromophenol blue, calculated using the following equation:

(1) $$\mathrm{Binding}\;\mathrm{amount}\;\mathrm{of}\;\mathrm{bromophenol}\;\mathrm{blue}\;(\mu g)\;=\;\frac{A_{\mathrm{Blank}}-A_{\mathrm{Sample}}}{A_{\mathrm{Blank}}}\times 200$$

A_Blank_ is the absorbance value of the control at 595 nm and A_Sample_ is the absorbance value of the sample at 595 nm.

#### 2.2.5. Protein Thermal Shift Assay

A protein thermal shift assay was performed to assess the thermal stability of the myofibrillar protein using a Real-Time PCR instrument (StepOnePlus, Thermo Fisher Scientific, Marsiling, Singapore), according to the manufacturer’s instructions. Myofibrillar protein was extracted from frozen surimi as described in Section 2.2.1. Subsequently, surfactant was added to 1 mg/mL of myofibrillar protein. The final reaction volume was 20 μL, consisting of 2 μL of 50× SYPRO orange dye, 2 μL of 1 mg/mL protein sample, and 16 μL of 20 mM PBS. The temperature was increased continuously from 25.0 °C to 95.0 °C at a ramp rate of 1%. After the protein thermal shift assay was completed, the software exported a data file containing the temperature data, fluorescence intensity-normalized data, and first derivative data for each well. Subsequently, curves of fluorescence intensity versus temperature were generated using OriginPro 2026 software.

### 2.3. Forward-Extrusion Behavior of Raw Surimi

**Figure 1:**
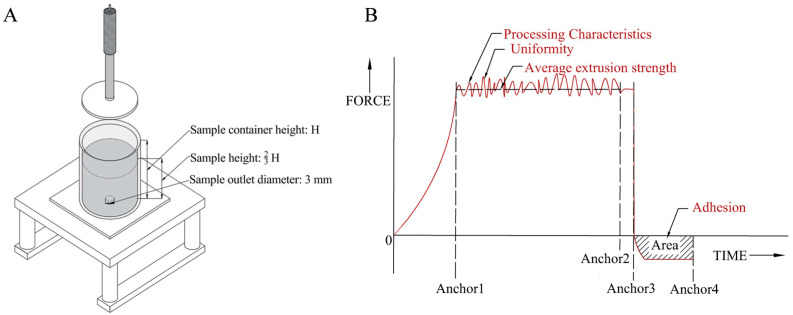
Illustration of the experimental set-up (**A**) and the typical test curve (**B**) of forward extrusion.

Raw surimi was loaded into the extrusion cell (cylinder container with a 3 mm diameter bottom opening) until it filled two-thirds of the cell, taking care to avoid entrapping air. A texture analyzer (TA-XT Plus, Stable Micro Systems Ltd., Godalming, UK) equipped with a plunger was used to extrude the surimi through the bottom opening, as illustrated in Figure 1A. The extrusion parameters were set as follows: pre-test speed of 1.0 mm/s, test speed of 1.0 mm/s, post-test speed of 10.0 mm/s, and trigger force of 50 g. The force during forward extrusion was recorded, and the average extrusion strength, processing characteristics, uniformity, and adhesion were calculated from the force–time curve (Figure 1B) according to the manufacturer’s instructions.

According to the macro program, four anchors were placed to define specific regions of the force–time curve. The first anchor was set at 0.5 s after the initial positive compression peak, and the second anchor was set at a plunger distance of 19 mm. The third anchor was positioned at the point where the force returned to zero after extrusion, and the fourth anchor was positioned at the maximum recording time. Based on these defined regions, the average extrusion strength was calculated as the sum of all the Y-axis data values between the first and second anchors divided by the total number of data points in the selected region. The processing characteristics were defined as the average drop in force between consecutive peaks and troughs within the same region. Uniformity was defined as the path length of an imaginary line joining all points in the selected region (between the first anchor and the second anchor). Adhesion was calculated as the negative area of the force–time curve from the third anchor (zero force) to the fourth anchor (maximum time).

### 2.4. Characterization of Surimi Gel

#### 2.4.1. Preparation of Surimi Gel

The thawed surimi was homogenized with a S18-LA 170 food processor (Joyoung Co., Ltd., Jinan, China) for 3 min and then the moisture content was adjusted to 80%. Subsequently, 2.0% (*w*/*w*) salt and 0.1% or 0.5% (*w*/*w*) Tween 20 or Tween 80 were added to the surimi. The control group was prepared without any emulsifier. The mixture was then homogenized for an additional 5 min to form a paste. The surimi paste was then filled into plastic casings (38 mm diameter) and subjected to a two-stage heat treatment: first, incubation at 40 °C for 60 min, followed by heating at 90 °C for 30 min to form the final gel. The resulting gel samples were designated as Control, Tween 20-0.1%, Tween 20-0.5%, Tween 80-0.1%, and Tween 80-0.5%. Finally, all samples were cooled in iced water for 30 min and then stored at 4 °C for further analysis.

#### 2.4.2. Gel Strength

The surimi gels were trimmed into cylinders with a uniform height of 20 mm for texture analysis according to the method of Yu et al. [24]. Gel strength was measured using a TA-XT Plus texture analyzer (Stable Micro Systems Ltd., Godalming, UK) equipped with a P/5S cylindrical probe. The test parameters were set as follows: pre-test, test, and post-test speeds of 1.00 mm/s, a compression distance of 15.00 mm, and a trigger force of 5.0 g. Gel strength was calculated as the product of the breaking force and the breaking deformation.

#### 2.4.3. Texture Profile Analysis (TPA)

TPA was performed using a TA-XT Plus texture analyzer (Stable Micro Systems Ltd., Godalming, UK) equipped with a P/50 cylindrical probe according to the method of Zhang et al. [29]. Cylindrical samples with a height of 20 mm were taken. The measurement parameters were set as follows: the pre-test, test and post-test speeds were 1.00 mm/s and the compression ratio was 40%.

The cook loss was determined by weighing the surimi samples before (m_1_) and after (m_2_) the two-stage heat treatment according to Li et al. [30]. It was calculated as follows:

(2) $$\mathrm{Cook}\;\mathrm{loss}\;(\%)\;=\;\frac{\left(m_{1}-m_{2}\right)}{m_{1}}\times 100$$

The measurement of centrifugation loss refers to the method of Yuan et al. [31] with slight modifications. Gel slices (3.00 ± 0.05 g) were wrapped in three layers of filter paper and centrifuged at 8000× *g* for 15 min at 4 °C. The sample weights before (m_3_) and after (m_4_) centrifugation were used to calculate the centrifugation loss as follows:

(3) $$\mathrm{Centrifugation}\;\mathrm{loss}\;(\%)\;=\;\frac{{(m}_{3}-m_{4})}{m_{3}}\times 100$$

#### 2.4.4. Low-Field Nuclear Magnetic Resonance (LF-NMR)

The transverse relaxation time (T_2_) and water distribution in the protein gel were measured using a NMI20-060VJS-I LF-NMR analyzer (Suzhou Newmai Analytical Instrument Co., Ltd., Suzhou, China), following the method described by Wang et al. [32]. Cylindrical samples (20 mm in height) were prepared and equilibrated at 25 °C for 1 h prior to analysis. The following parameters were used for the pulse: a spectrometer frequency of 21 MHz, a spectral width of 200 kHz, pulse widths at 90 degrees of 7.52 μs, a waiting time of 1500 ms, and 4 scans.

### 2.5. Scanning Electron Microscopy (SEM)

The microstructure of the surimi gel was observed using a scanning electron microscope (Quanta FEG 250, FEI Co., Hillsboro, OR, USA), according to Htwe et al. [33] and Shi et al. [34]. Gel samples were cut into 3 mm × 3 mm × 1 mm cubes and incubated in 2.5% glutaraldehyde at 4 °C for 3 h. After fixation, the samples were rinsed with distilled water and dehydrated through a graded ethanol series (30, 50, 70, 90, 95, and 100%), for 15 min at each concentration. The ethanol was then replaced by immersing the samples in a tert-butanol/ethanol mixture (1:1, *v*/*v*) followed by pure tert-butanol. The dehydrated samples were freeze-dried for 48 h and then the dried samples were mounted on aluminum stubs. The samples were observed at 20 kV acceleration voltage.

### 2.6. Statistical Analysis

Statistical analyses were performed using one-way analysis of variance (ANOVA), followed by Tukey’s HSD for post hoc comparisons, using SPSS software (version 20.0). Differences were considered statistically significant at *p* < 0.05. All experimental factors were considered fixed factors.

## 3. Results and Discussion

### 3.1. Characterization of Protein in the Raw Surimi

**Figure 2:**
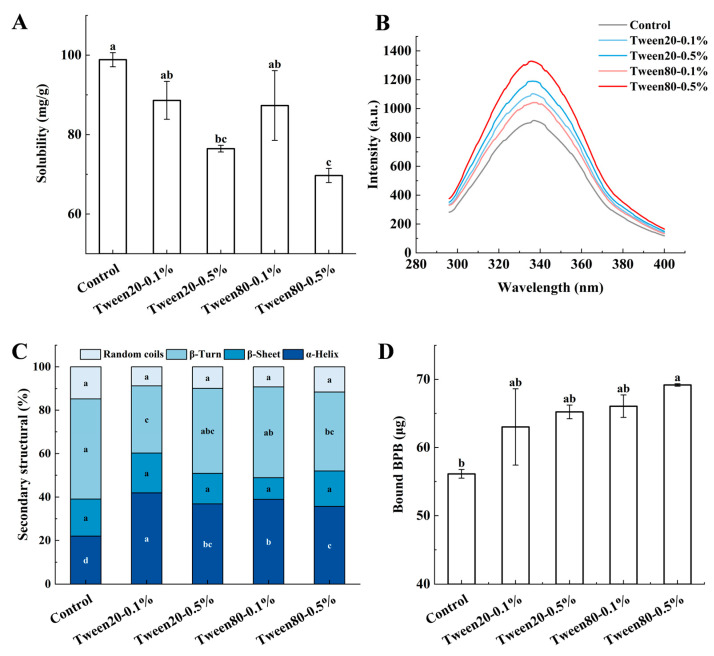
Effects of added emulsifiers (Tween 20 or Tween 80) on the protein extractability (**A**), intrinsic fluorescence intensity (**B**) and secondary structure (**C**) and surface hydrophobicity (**D**) of surimi gel. Different letters in the same index indicate significant differences (*p* < 0.05).

Solubilization of myofibrillar proteins from the sarcomeric structure is critical for the heat-induced gelation of surimi. According to Wang et al. [35], Tween 20 and Tween 80 can directly interact with proteins and disrupt the delicate balance of all the folding forces responsible for the protein conformational stability. As shown in Figure 2A, the solubility of myofibrillar proteins significantly decreased with the addition of Tween 20 or Tween 80 and declined further as the concentration of the added surfactant increased. At the level of 0.5%, Tween 80 appeared to have stronger effects in lowering protein solubility than Tween 20, likely due to the more hydrophobic chain of Tween 80. In the manufacturing of surimi gel, chopping and salting are essential processing steps. Both chopping and salting lead to protein denaturation, as observed in our previous studies [36,37]. Fluorescence intensity can reflect protein denaturation by evaluating changes in the surrounding environment of tryptophan chromophores [38]. The addition of Tween 20 or Tween 80 resulted in a significantly higher fluorescence intensity (Figure 2B), indicating that the native protein structure was better preserved after chopping and salting. In contrast, Xia et al. [39] found that the surfactant lecithin led to a decrease in the intrinsic fluorescence intensity of myofibrillar proteins. Other than the chemical nature of surfactants, the way they were added to the proteins was different. In this study, surfactants were added before chopping; therefore, they may exert a protective effect on protein against mechanical stress-induced denaturation. One typical feature of myofibrillar protein denaturation is the loss of α-helix structure [40]. The protective effect of Tween 20 or Tween 80 on protein denaturation was also supported by Raman spectroscopy analysis, in which a higher proportion of α-helix structure was observed in the presence of Tween 20 or Tween 80 (Figure 2C). Similarly, Chou et al. [19] found that Tween 20 and Tween 80 protected albutropin against agitation-induced aggregation and suggested that the protection effect was due to increased free energy of unfolding caused by binding of Tween 20 or Tween 80. Hillgren et al. [41] showed that surfactant-induced denaturation of lactate dehydrogenase occured only in the case of ionic SDS but not in nonionic Tween 80. Although the proteins were less denatured in the presence of added surfactant, their ability to bind hydrophobic BPB increased (Figure 2D). This phenomenon may be attributed to the binding of Tween 20 or Tween 80 to the hydrophilic regions on the protein surface, thereby increasing the net surface hydrophobicity. According to Kumar et al. [20], the binding of surfactants at low concentrations may not cause protein denaturation. However, it may provide steric hindrance by decreasing the intermolecular contacts that precede the onset of gelation.

**Figure 3:**
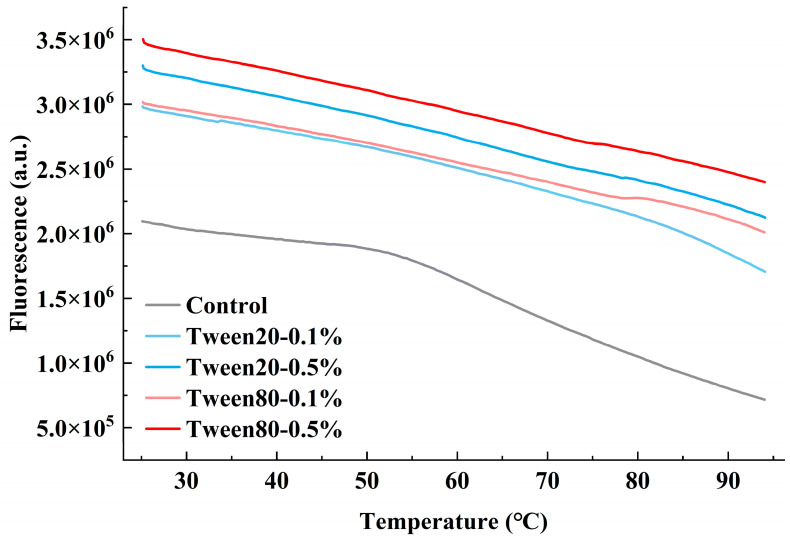
Thermal shift assay of myofibrillar protein with Tween 20 or Tween 80 added.

A protein thermal shift assay was performed to assess the thermal stability of the myofibrillar protein (Figure 3). According to Rosa et al. [42], as the dye/protein mixture was heated, the protein unfolded, exposing its hydrophobic core. The dye bound to this hydrophobic environment, leading to an increase in fluorescence intensity. Upon further heating, the unfolded protein chains aggregated, excluding the dye. The released dye returned to the aqueous environment, causing the fluorescent signal to decrease. In the presence of surfactant, the fluorescence intensity was significantly stronger than the myofibrillar protein alone, likely due to the increased surface hydrophobicity (Figure 2D) caused by the binding of the surfactant molecules to the hydrophilic area of the protein surface. During heating, the fluorescence intensity continued to decrease, indicating progressive aggregation of myofibrillar proteins. After the temperature exceeded 50 °C, the decrease in the fluorescence intensity became faster in the control group, while the other groups did not show such drastic changes, suggesting that the presence of surfactants hindered the aggregation of myofibrillar proteins. Similar effects for Tween 80 in preventing protein denaturation have been reported in pharmaceutical studies [19,35,43].

### 3.2. Forward Extrusion

The flow property of surimi pastes determines the ability to pump the material within the manufacturing plant and affects the extrusion properties of the material [44,45]. Rheological measurements can give insight into the interactions between myofibrillar proteins without extraction, and extraction with high ionic strength may lead to changes in the original state of the myofibrillar proteins in the fiber fragments [46]. Extrusion tests can be used to analyze viscous food materials, such as meat batter [47], pea protein paste [48] and bread batter [49]. However, these studies mainly focused on the firmness of the batter, while more information about the food properties can be obtained. In this study, forward extrusion has been applied to evaluate surimi properties. As shown in Table 1, the addition of Tween 20 and Tween 80 led to a significantly higher value for the average extrusion strength. According to Hamm [46], the rheological behavior of minced meat depends on properties of the suspended particles and the viscosity of the liquid phase, which is influenced by the amount and type of dissolved proteins. The processing characteristics were found to be higher with the addition of Tween 20 and Tween 80 in a dose-dependent manner. This suggested the cohesion of the surimi was weakened by Tween 20 or Tween 80 addition. Similarly, Malhotra and Coupland [50] found that in soy protein isolate, the viscosity of the Tween 20-containing samples was low, while the SDS-containing samples were much more viscous. Addition of Tween 20 and Tween 80 improved uniformity, indicating the surimi can be smoothly extruded. At the same addition level of 0.1%, Tween 80 was more effective than Tween 20, suggesting the importance of the hydrophobicity of surfactants. The adhesion of the surimi paste was reduced, likely due to the coating of surfactants on the surimi particles.

### 3.3. Characterization of Surimi Gel

Protein–surfactant interactions can be used to direct heat-induced protein gelation [20]. Kumar and Aswal [51] demonstrated that temperature-driven gelation of BSA was dependent on the type and the concentration of surfactants. In this study, the incorporation of Tween 20 or Tween 80 led to a lower gel strength, and further addition of the surfactants did not cause significant changes (Table 2). Hardness and gumminess showed a similar pattern. In general, only a marginal difference in chewiness, resilience, cohesion, and springiness were found among the groups. In contrast, addition of Tween 20 or Tween 80 led to a dose-dependent decrease in adhesiveness. This can be expected due to the presence of surfactants, and it was similar to the adhesion of raw surimi during forward extrusion (Table 1). Murugesan et al. [52] investigated the effect of saponin (a natural surfactant) fortification on the gelling properties of surimi from Threadfin Bream and found that elastic modulus of the gel increased at a low dose, but decreased at a high dose. Despite the fact that strong mechanical properties are traditionally favored by the surimi industry, the weakened gel strength following addition of Tween 20 or Tween 80 may be well suited for the development of foods for the elderly. Recently, Zhu et al. [53] aimed to optimize the properties of surimi gels to enhance their suitability for individuals with dysphagia. Principal component analysis identified that gel strength was one of the key parameters affecting swallowing function, indicating that surimi gels for dysphagia patients should exhibit low breaking force and breaking distance. If needed, the gel strength of surimi can be modulated through various ingredients [34,54,55] or processes [3,26,56].

### 3.4. Water-Holding Properties

**Figure 4:**
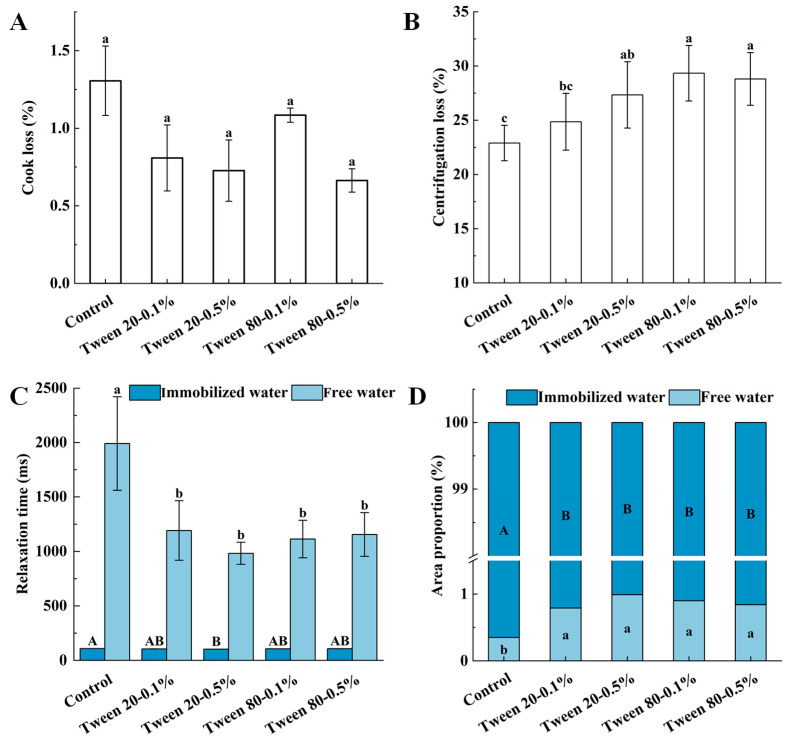
Effects of added emulsifiers (Tween 20 or Tween 80) on the cook loss (**A**), centrifugation loss (**B**), water relaxation time (**C**), and relative content of different states in water (**D**) of surimi gel. Different letters in the same index indicate significant differences (*p* < 0.05).

Methods for measuring and characterizing the water-holding capacity (WHC) of food protein gels span three interconnected dimensions: macro-quantitative determination, microscopic structural visualization, and molecular-scale water dynamics tracking [57]. As shown in Figure 4A,B, cook loss and centrifugation loss were determined to evaluate the WHC of the surimi gels. Cook loss showed a decreasing trend with the addition of 0.1% or 0.5% Tween 20 and 0.5% Tween 80. However, centrifugation loss increased in gels containing 0.5% Tween 20 or either level of Tween 80 (0.1% and 0.5%), indicating that a larger portion of water is loosely bound in the surimi gel with added surfactants. This is confirmed by the NMR results where the proportion of free water is greater in surimi gels with added surfactants. This can be partly explained by the increased protein surface hydrophobicity (Figure 2). The significant reduction in the relaxation time corresponding to free water suggested that this fraction of water is more tightly bound to the gel matrix, but not strong enough to withstand the centrifugal force as applied in this study. The results were in line with Chen et al. [58] where the WHC of the emulsion gels decreased with increased content of Tween 20.

### 3.5. SEM

**Figure 5:**
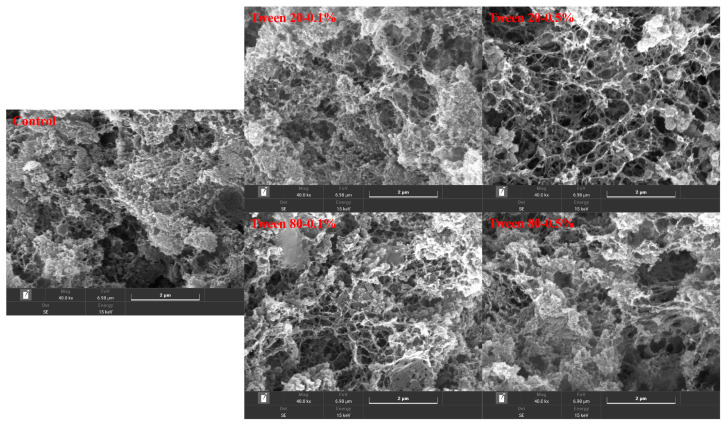
Representative SEM images of surimi gel incorporated with Tween 20 or Tween 80.

As shown in Figure 5, SEM images revealed that all surimi gels possessed porous structures. The control gel exhibited the smallest pore size, whereas the addition of Tween 20 or Tween 80 increased the porosity. The gel with 0.5% Tween 20 incorporated displayed the largest pores. These results indicate both Tween 20 and Tween 80 hindered the formation of a continuous gel network and led to a less compact microstructure. This is consistent with the findings of Luo et al. [59], who reported that oil droplets coated with Tween 80 acted as inactive fillers, which flocculated and destroyed the protein gel matrix, thereby creating larger pores in the network. In this study, no oils were added to the surimi and therefore the added Tween 20 or Tween 80 very easily bound to the myofibrillar proteins. This binding led to a reduced solubility of the myofibrillar proteins and hence less available gelling agents in the surimi paste. The binding of Tween 20 or Tween 80 may reduce the interaction among myofibrillar proteins. Wang et al. [60] reported that the gelation of egg yolks during freezing is associated with the breakdown of lipoproteins and the subsequent interactions among protein molecules.

### 3.6. Proposed Mechanism for the Effect of Tween Addition on Surimi Properties

**Figure 6:**
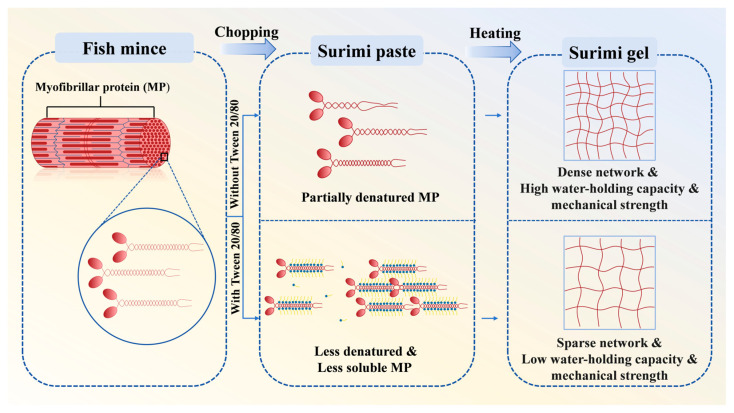
A proposed mechanism of how the added surfactant (Tween 20 or Tween 80) affects the surimi gel properties.

In the present study, we investigated the effects of Tween 20 or Tween 80 on the physicochemical properties of surimi gels. Results showed that addition of Tween 20 or Tween 80 reduced the solubility of the myofibrillar proteins (Figure 2A), but the protein was less denatured after chopping (Figure 2B,C), although the surface hydrophobicity increased (Figure 2D). A forward-extrusion test indicated that the surimi paste was less coherent and more easily extruded. After gelling, the gel strength and hardness of surimi gel showed significant reduction in the presence of Tween 20 or Tween 80 (Table 2), while the water-holding capacity was also reduced (Figure 4A,B). Due to the presence of the surfactant, both the surimi paste (Table 1) and the surimi gel (Table 2) were less adhesive. Based on the above-mentioned experimental results, a mechanism of how the added surfactant (Tween 20 or Tween 80) affects the surimi gel properties was proposed (Figure 6): During chopping, the surfactant binds to myofibrillar proteins and protects them against denaturation. However, the binding leads to increased surface hydrophobicity which promotes aggregation of the myofibrillar proteins and reduces their solubility. The reduced solubility of myofibrillar proteins essentially lowers the concentration of gelling compounds and therefore the formed gels exhibit a loose network, eventually leading to lower water-holding capacity and mechanical strength.

## 4. Conclusions

This study demonstrated that the addition of nonionic surfactants (Tween 20 and Tween 80) during the chopping process significantly modulates the physicochemical properties of myofibrillar proteins and the resulting surimi gel. During chopping, Tween 20 and Tween 80 exert a protective effect on proteins, effectively mitigating denaturation as evidenced by higher intrinsic fluorescence intensity and a greater proportion of α-helix structures. Furthermore, the surfactants improve the flowability and uniformity of the surimi paste while reducing its cohesion and adhesion, which is beneficial for industrial extrusion and pumping processes. However, the binding of these surfactants to myofibrillar proteins leads to a dose-dependent decrease in protein solubility, which subsequently interferes with the formation of a continuous and compact protein network. Consequently, the heat-set gels with Tween 20 or Tween 80 incorporated exhibit a more porous microstructure, resulting in reduced gel strength, hardness, and water-holding capacity. While these surfactants weaken the mechanical properties, they simultaneously reduce the adhesiveness of the gel. Therefore, Tween 20 and Tween 80 can be utilized as effective additives to tailor the texture of surimi products, offering promising potential in the development of specialized, easy-to-swallow foods for the elderly.

## Figures and Tables

**Table 1:** Effects of added surfactants (Tween 20 or Tween 80) on the behavior of surimi gel during forward extrusion.

Group	Average Extrusion Strength/kg	Processing Characteristics/g	Uniformity/kg∙s	Adhesion/kg∙s
Control	2.02 ± 0.40 ^b^	10.74 ± 1.35 ^c^	14.91 ± 2.40 ^a^	−1.08 ± 0.11 ^b^
Tween 20-0.1%	2.97 ± 0.31 ^a^	86.89 ± 22.25 ^bc^	9.61 ± 2.22 ^b^	−0.69 ± 0.04 ^a^
Tween 20-0.5%	2.81 ± 0.21 ^a^	152.56 ± 35.32 ^ab^	3.98 ± 0.66 ^c^	−0.68 ± 0.13 ^a^
Tween 80-0.1%	3.06 ± 0.03 ^a^	58.52 ± 17.36 ^bc^	2.37 ± 0.06 ^c^	−0.77 ± 0.11 ^a^
Tween 80-0.5%	2.85 ± 0.15 ^a^	215.25 ± 52.89 ^a^	3.68 ± 0.93 ^c^	−0.85 ± 0.17 ^b^

^a–c^ Different superscript letters in the same column indicate significant differences at *p* < 0.05.

**Table 2:** Effects of added surfactants (Tween 20 or Tween 80) on the gel strength and TPA attributes of surimi gel.

Group	Gel Strength/kg∙mm	Hardness/kg	Gumminess/kg	Chewiness/kg	Resilience/%	Cohesion/%	Springiness/%	Adhesiveness/g∙s
Control	3.34 ± 0.56 ^a^	1.41 ± 0.05 ^a^	1.24 ± 0.04 ^a^	1.19 ± 0.04 ^a^	61.63 ± 0.76 ^a^	0.88 ± 0.01 ^a^	95.89 ± 0.31 ^b^	−109.67 ± 11.31 ^b^
Tween 20-0.1%	2.81 ± 0.33 ^ab^	1.34 ± 0.07 ^ab^	1.18 ± 0.06 ^ab^	1.13 ± 0.07 ^a^	60.67 ± 069 ^a^	0.878 ± 0 ^a^	95.72 ± 1.74 ^b^	−83.01 ± 9.55 ^ab^
Tween 20-0.5%	3 ± 0.36 ^ab^	1.29 ± 0.04 ^b^	1.14 ± 0.04 ^b^	1.11 ± 0.04 ^a^	61.52 ± 0.94 ^a^	0.883 ± 0.01 ^a^	97.67 ± 1.01 ^a^	−43.55 ± 33.36 ^a^
Tween 80-0.1%	2.98 ± 0.24 ^ab^	1.33 ± 0.08 ^ab^	1.17 ± 0.06 ^ab^	1.13 ± 0.06 ^a^	61.66 ± 0.37 ^a^	0.884 ± 0.01 ^a^	96.13 ± 0.59 ^ab^	−81.91 ± 30.99 ^ab^
Tween 80-0.5%	2.67 ± 0.24 ^b^	1.31 ± 0.05 ^ab^	1.16 ± 0.05 ^b^	1.12 ± 0.04 ^a^	61.14 ± 0.55 ^a^	0.883 ± 0.01 ^a^	97.07 ± 0.77 ^ab^	−64.16 ± 31.73 ^a^

^a,b^ Different superscript letters in the same column indicate significant differences at *p* < 0.05.

## Data Availability

The original contributions presented in this study are included in the article. Further inquiries can be directed to the corresponding author.
